# NF-kB-dependent activation of STAT3 by *H. pylori* is suppressed by TFF1

**DOI:** 10.1186/s12935-021-02140-2

**Published:** 2021-08-21

**Authors:** Mohammed Soutto, Nadeem Bhat, Shayan Khalafi, Shoumin Zhu, Julio Poveda, Monica Garcia-Buitrago, Alexander Zaika, Wael El-Rifai

**Affiliations:** 1 Department of Surgery, Miller School of Medicine, University of Miami, Miami, FL USA; 2 Department of Veterans Affairs, Miami Healthcare System, Miami, FL 33136-1015 USA; 3 Department of Pathology, Miller School of Medicine, University of Miami, Miami, FL USA

**Keywords:** TFF1, *Helicobacter pylori*, NFκB, STAT3, Gastric cancer, *H. pylori*-induced, Inflammation

## Abstract

**Background:**

*H. pylori* infection is the main risk factor for gastric cancer. In this study, we investigated *H. pylori*-mediated activation of STAT3 and NF-κB in gastric cancer, using in vitro and in vivo models.

**Methods:**

To investigate the activation of NF-κB and STAT3 by *H. pylori* strains we used in vitro and in vivo mouse models, western blots, immunofluorescence, ChIP Assay, luciferase and quantitative real-time PCR assays.

**Results:**

Following infection with *H. pylori* in vitro, we found an earlier phosphorylation of NF-kB-p65 (S536), followed by STAT3 (Y705). Immunofluorescence, using in vitro and in vivo models, demonstrated nuclear localization of NF-kB and STAT3, following *H. pylori* infection. NF-kB and STAT3 luciferase reporter assays confirmed earlier activation of NF-kB followed by STAT3. In vitro and in vivo models demonstrated induction of mRNA expression of *IL-6* (p < 0.001), *VEGF-α* (p < 0.05), *IL-17* (p < 0.001), and *IL-23* (p < 0.001). Using ChIP, we confirmed co-binding of both NF-kB-p65 and STAT3 on the *IL6* promoter. The reconstitution of Trefoil Factor 1 (TFF1) suppressed activation of NF-kB with reduction in IL6 levels and STAT3 activity, in response to *H. pylori* infection. Using pharmacologic (BAY11-7082) and genetic (IκB super repressor (IκBSR)) inhibitors of NF-kB-p65, we confirmed the requirement of NF-kB-p65 for activation of STAT3, as measured by phosphorylation, transcription activity, and nuclear localization of STAT3 in in vitro and in vivo models.

**Conclusion:**

Our findings suggest the presence of an early autocrine NF-kB-dependent activation of STAT3 in response to *H. pylori* infection. TFF1 acts as an anti-inflammatory guard against *H. pylori*-mediated activation of pro-inflammatory networks.

**Supplementary Information:**

The online version contains supplementary material available at 10.1186/s12935-021-02140-2.

## Background

The development of gastric cancer is a multifactorial process, including environmental, host-related and dietary factors [1]. *Helicobacter pylori (H. pylori)*, a gram-negative bacterium, has been considered an important environmental risk factor for gastric cancer, classified by the World Health Organization as class I carcinogen [2, 3]. It is estimated that half of the world’s population is infected by this bacteria [4, 5]. *H. pylori* strains contain a virulence determinant region known as cag Pathogenicity Island (cag-PAI) where CagA (cytotoxin-associated gene) is one of the 30 genes encoded by cag-PAI [6]*.* CagA is translocated from bacteria into human epithelial cells through a type IV secretion system and controls cellular effects through regulation of specific signal transduction pathways [7]. *H. pylori* initiates a series of histological changes called the Correa’s cascade that includes progression from chronic gastritis to chronic atrophic gastritis, intestinal metaplasia, atypical hyperplasia and ultimately gastric cancer[8, 9]

Chronic infection with *H. pylori* plays an important role in activating several pro-inflammatory signaling [10]. NF-κB and STAT3 are among the major pro-inflammatory pathways activated during carcinogenesis [11]. NF-κB is a transcriptional factor which is constitutively activated in gastric cancer [12], and its activation is mediated via cagA after *H. pylori* infection [13]. Activation of NF-κB induces release of several pro-inflammatory cytokines and chemokines that are involved in proliferation, angiogenesis, invasion and blockade of apoptosis [14]. STAT3 is another transcriptional factor that is activated by cytokines and growth factor-induced signaling [15]. A number of recent reports demonstrated functional interaction between NF-κB and STAT3 transcriptional activities for maximum induction and activation of cytokines [16].

TFF1 is a small secreted protein that protects the integrity of the gastric mucosa and promotes its repair after injury [17]. Downregulation of TFF1 expression occurs in more than half of gastric adenocarcinomas via a number of different molecular mechanisms including loss of heterozygosity, mutations, deletions, promoter hypermethylation, or transcription regulation [18–22]. In previous studies, we and others have demonstrated that TFF1 plays the role of a typical model of tumor suppressor gene [23, 24]. Several studies indicated that TFF1 is a tumor suppressor gene with pro-apoptotic, anti-proliferative, and anti-inflammatory functions [23, 25–27].

In this study, we investigated the role of TFF1 in suppressing *H. pylori*-mediated activation of NF-κB and STAT3 using in vitro and in vivo gastric neoplasm models. Our results suggest that activation of NF-κB precedes STAT3 activation, in response to *H. pylori* infection. We also found that STAT3 activation may be dependent on NF-κB. TFF1 protects against *H. pylori*-mediated activation of NF-κB and STAT3.

## Material and methods

### Reconstitution of TFF1 expression in cell lines

The gastric cancer cell lines AGS and SNU-1 were purchased from ATCC (American Tissue Culture Collection, Manassas, VA). HGC-27 were purchased from AMSBIO (Amsbio, United Kingdom). Cells were maintained in Ham’s F-12 with 10% fetal bovine serum (Invitrogen Life Technologies) and incubated at 37 °C in 5% CO_2_. The establishment of AGS cells stably-expressing human TFF1 was described before [23]. Briefly, the human *TFF1* coding sequence was amplified by PCR, and cloned in-frame into the mammalian expression vector pcDNA3.1 (Invitrogen). Using Fugene-6 (Cat# E2691 Roche Applied Science) and following the manufacturer’s protocols, AGS cells were transfected with pcDNA3.1-TFF1 or pcDNA3.1 empty vector as a control. Stable cell lines were selected using G418 (Cat# 10131035 Invitrogen) (0.5 mg/ml). For the transient expression of TFF1, SNU-1 cells were transfected with the mammalian expression plasmid, pTT5 [28] in frame with TFF1, or PTT5 empty vector using Fugene-6 for 48 h. Protein expression of TFF1 in these cells was confirmed by using TFF1 Antibody (Origene, Rockville, MD).

### *H. pylori* bacterial culture

J166 and 7.13 are two *H. pylori* strains, *CagA* + types. Both were used for the in vitro studies. J166 *H. pylori* is a clinical isolate of human-derived *H. pylori* [29], and 7.13 *H. pylori* is derived through in vivo adaptation of a clinical *H. pylori* strain, B128 [30]. For the mouse infection, we utilized the pre-mouse Sydney strain 1 (PMSS1). PMSS1 is the wild-type rodent-adapted c*ag* + *H. pylori* strain derived from the parental strain of the mouse Sydney strain (SS1), acquired form a clinical isolate of a duodenal ulcer patient [31]. All the *H. pylori* strains were a gift from Dr. Peek at Vanderbilt University. The cultures of *H. pylori* were made on Brucella agar (BBL/Becton Dickinson, Sparks, MD) supplemented with 5% heat-inactivated BSA (Invitrogen) and a combination of antibiotics (vancomycin cat#1404-93-9, 100 µg/ml; bacitracin cat# 1405-87-4, 200 µg/ml; amphotericin B cat#1397-89-3, 20 µg/ml; nalidixic acid cat#389-08-2, 10.7 µg/ml; polymyxin B cat#1405-20-5, 3.3 µg/ml) (Sigma-Aldrich, St. Louis, MO). *H. pylori* cultures from inoculation were grown in Brucella broth supplemented with 5% BSA and vancomycin antibiotic. After 24 h, bacteria were pelleted and resuspended in Brucella broth.

### Animals studies and drug administration

All animal procedures were approved by the Animal Care Committee of University of Miami. Three groups, each of 8–10 TFF1-KO mice, were used. Two groups were inoculated for three consecutive days by oral gavage with 1 × 10^9^ colony-forming units of *H. pylori* PMSS1 strain in 0.5 ml of Brucella broth, as described before [32]. One groups was given Brucella broth alone and used as a control. After two weeks, one group of PMSS1 infected mice received BAY 11–7082 (Cat# 196870 Calbiochem, San Diego, CA) (5 mg/Kg) through intraperitoneal injection, once every 3 days. Un-infected and *H. pylori* infected mice were used as control groups, receiving injection with 1xPBS. Mice were euthanized a day after the last injection. Frozen tissues and formalin fixed paraffin-embedded stomach tissue samples were used for RNA isolation and histology.

### Immunofluorescence procedure

AGS cells stably expressing TFF1 or empty vector pcDNA (control) were seeded in 8-well chambers. After 48 h, the cells were infected with *H. pylori* 7.13 strain (100:1) for 24 h. The immunofluorescence technique was performed as described before [33]. Cells were then washed with PBS and fixed with fresh 4% paraformaldehyde solution for 15 min at room temperature. Cells were washed twice with PBS and incubated for 20 min in blocking solution goat serum (Zymed Laboratories, San Francisco, CA) at room temperature in a humidified chamber. Next, cells were incubated in the specific primary monoclonal rabbit-antibody p-STAT3 (Y705) (Cat# ab76315, Abcam, Cambridge, MA) and primary monoclonal antibody mouse p-NF-κB-P65 (Cat# S536) (Cat# sc-136548 Santa Cruz Biotechnology, CA) diluted in blocking buffer (1:200) overnight at 4 °C. The following day, chamber slides were washed three  times in PBS and incubated for 1 h with secondary antibodies goat anti-rabbit IgG conjugated to fluorophore Alexa Fluor 488 (Cat# A-11034, Invitrogen) and goat anti-mouse IgG conjugated to Alexa Fluor 568 (Cat# A-11004, Invitrogen) diluted in blocking buffer (1:500). The cells were then washed in PBS, mounted with Vectashield/DAPI (Vector Laboratories, Burlingame, CA), and visualized using an inverted microscope (KEYENCE BZ-X700, Osaka, Japan; magnification 40). Using ImageJ software (http://www.uhnresearch.ca/facilities/wcif/imagej/), a total of 200 cells from each experiment were counted with an automatic particle counting after setting an automatic threshold range. The image was transformed into a binary image and the total number of cells in each field were counted using watershed separation. The percentage of nuclear NF-κB and STAT3 positive cells were calculated as the number of cells showing green or red nuclear immunostaining, respectively, divided by the total cell number showing DAPI (blue) nuclear staining × 100.

Mouse tissues' immunofluorescence procedure was performed as described before [33]. Gastric tissues were collected and fixed in 10% formalin, embedded in paraffin and blocks were cut to 5 μm sections on glass slides. The tissue sections were deparaffinized and heated in a pressure cooker for 12 min in TE buffer to perform antigen retrieval. Sections were blocked with PBS containing 5% bovine serum albumin for 1 h at room temperature. The sections were incubated with primary rabbit-antibody p-STAT3 (Y705) and primary antibody mouse -p-NF-κB-P65 (S536) and diluted in blocking buffer (1:200) overnight at 4 °C. The next day, tissue sections were washed and incubated for 1 h at room temperature with secondary antibodies; goat anti-rabbit IgG and conjugated to fluorophore Alexa Fluor 488 and goat anti-mouse IgG conjugated to Alexa Fluor 568 (Invitrogen) diluted in blocking buffer (1:500). Sections were washed three times and mounted with Vectashield/DAPI. Sections were imaged using an  inverted microscope (KEYENCE BZ-X700, Osaka, Japan; magnification 40), using Z-stack.

### Luciferase reporter assay

To measure the transcription activity of NF-κB and STAT3 signal transduction pathway, we used the NF-κB-Luc (Cat# 631743) from (Clontech, Palo Alto, CA) and STAT3-Luc reporter vector (Cat# 8688) from (Addgene, Cambridge, MA). Cells were seeded in 24-well plates. The next day, cells were transiently transfected with 500 ng of NF-κB-Luc or STAT3–Luc and 250 ng of β-galactosidase, as a control plasmid, using FuGENE 6 according to the manufacturer's instructions (Roche Applied Science). Cells were incubated for 24 h and then infected with *H. pylori* J166 or 7.13 strain. Luciferase and β-galactosidase activities were measured after 4 h (NF-κB-Luc) or 24 h (STAT3-Luc). These time points were selected based on our experiments that showed differences in the onset of activation where NF-κB activation preceded STAT3 activation. The firefly luciferase activity was normalized to β-galactosidase activity.

### Western blotting

RIPA buffer, a cocktail of protease inhibitors and phosphatase inhibitors (Pierce, Rockford, IL, USA), was used as lysis buffer. Cell lysates were centrifuged at 3500 r.p.m. for 10 min at 4 °C. Protein samples (10–15 μg) were run on SDS/polyacrylamide gel electrophoresis (PAGE) and transferred onto nitrocellulose membranes. Specific monoclonal antibodies against phospho-STAT3 (Y705) (Cat# 9145), phospho-NF-κB-P65 (S536) (Cat# 3033), total STAT3 (Cat# 12640), total NF-κB-P65 (Cat# 8242) and β-Actin (Cat# 4970) and were obtained from Cell Signaling (Cell Signaling Technology, Beverly, MA). The monoclonal antibody against Rabbit anti-*H. pylori* Cag (Cat# HPM-5001-5) antigen IgG was obtained from Austral Biologicals (San Ramon, CA). For nuclear and cytoplasmic protein fractions, we used NE-PER Nuclear and Cytoplasmic Extraction Reagents (Pierce Biotechnology Inc.), following the manufacturer’s instructions. The nuclear and cytoplasmic protein fractions were normalized to anti-P84 monoclonal antibody (Cat# ab131268) (Abcam, Cambridge, MA) and β-Tubulin (Cat# 2146) (cell signaling), respectively. All antibodies were used at 1:1000 dilution.

### Real-time quantitative RT-PCR (RT-qPCR)

For gene expression, we used the RNeasy Mini kit (Qiagen, Germantown, MD) for total RNA extraction and iScript cDNA Synthesis Kit (Bio-Rad, Hercules, CA) for cDNA synthesis. For mouse and human primers design, we used the online software Primer 3 (http://frodo.wi.mit.edu/primer3/). The forward and reverse primers' sequences were designed to span exon-exons junctions for each gene (human: *IL-6, VEGF-α, IL-17* and *IL-23;* mouse: *Il-6, Vegf-α, Il-17* and *Il-23*) (Table 1). All primers were obtained from IDT (Integrated DNA Technologies, Coralville, IA). The RT-qPCR was performed in CFX connected Real-Time system from BIO-RAD using iQ™ SYBR® Green supermix. The reactions were carried on in triplicates and the threshold cycle numbers were averaged. The results of the genes' expression were normalized to the *HPRT* housekeeping gene HPRT. The calculation of the expression ratios was done according to the 2^(Rt–Et)^/2^(Rn–En)^ formula, where the threshold cycle number for the reference gene observed in the test samples is “Rt”and the experimental gene observed in the test samples is “Et”. The reference gene observed in the reference samples is “Rn” and the experimental gene observed in the reference samples is “En”. Rn and En values were calculated as an average of all reference samples [34].

**Table 1:** Oligonucleotide sequence of human and mouse RT-qPCR primers

Gene ID	Human Forward Primers	Human Reverse Primers	Size (bp)
IL-6	CCCTGAGAAAGGAGACATGTAA	TCTTTTTCAGCCATCTTTGGA	129
VEGF-α	CCTCCGAAACCATGAACTTT	ATGATTCTGCCCTCCTCCTT	122
IL-17	CTGTGTCACCCCGATTGTC	TTGAAGGATGAGGGTTCCTG	115
IL-23	AGAAGCTCTGCACACTGGC	CCACACTGGATATGGGGAAC	109

Gene ID	Mouse Forward Primers	Mouse Reverse Primers	
Il-6	GTTCTCTGGGAAATCGTGGA	GGAAGTTTCAGATTGTTTTCTGC	114
Vegf-α	GGAGAGCAGAAGTCCCATGA	TCGGGGTACTCCTGGAAGAT	150
Il-17	CAGGACGCGCAAACATGA	GCAACAGCATCAGAGACACAGAT	57
Il-23	GCACCTGCTTGACTCTGACA	ATCCTCTGGCTGGAGGAGTT	115

### ChIP assay

After *H. pylori* infection, AGS pcDNA and AGS-TFF1 cells were incubated with formaldehyde (Sigma-Aldrich) 1% final concentration to crosslink the protein-DNA complexes. Chromatin were sheared on ice by sonication for four cycles (30 s “ON”, 30 s “OFF” at 40% amplitude) to yield an average length of about 235 bp. ChIP assay was performed as previously described [33], using the Zymo-Spin ChIP Kit (Irvine, California, USA) and following the manufacturer’s protocol. Briefly, the supernatants of the fragmented lysates were diluted tenfold with chromatin dilution buffer. Chromatin solutions were immunoprecipitated with NF-κB (Cat# 8242) or STAT3 (Cat# 12640) monoclonal antibodies from cell signaling (1:100 dilution) at 4 °C overnight. ZymoMag Protein A beads were added to the lysate to isolate the antibody-bound complexes. The eluate was reverse cross-linked by heating at 65 °C for 30 min. Samples were then treated with proteinase K for 90 min at 65 °C to digest the proteins that were immunoprecipitated. The final eluate is purified DNA, which was analyzed by RT-qPCR  for NF-κB or STAT3 binding to the promoter sequences of *IL6* target gene, and normalized to 3% of the input. Validated primers from previous studies were used, and their sequences were: (-167) IL6-CHIP-NF-κB-F: 5′-CCTCACCCTCCAACAAAGAT-3′ and (-114) IL6-CHIP-NF-κB -R: 5′-TTG AGA CTC ATG GGA AAA TCC-3′ [35]; (-143) IL6-CHIP-STAT3-F: 5′-GTT GTG TCT TGC CAT GCT AA A G-3′ and (48) IL6-CHIP-STAT3-R: 5′-AGA ATG AGC CTC AGA CAT CTC C-3′ [36]. The control primers were designed 500 base pairs upstream of STAT3 binding sites on the IL6 promoter; the sequences of the control primers were CTRL-F: 5′-GAG AAA GGA GGT GGG TAG GC-3′ and CTRL-R: 5′- AAA AGG AAG CCC TGA GAA GC-3′.

### Statistical analysis

Using GraphPad Prism software, a One-way ANOVA Newman-Keuls Multiple Comparisons Test was used to compare the differences between three or more groups. A two-tailed Student’s test was used to compare the statistical difference between two groups. The differences were considered statistically significant when the P value was P < 0.05.

## Results

### Reconstitution of TFF1 decreases *H. pylori*-mediated activation of NF-κB and STAT3 in gastric cancer cells

**Fig. 1:**
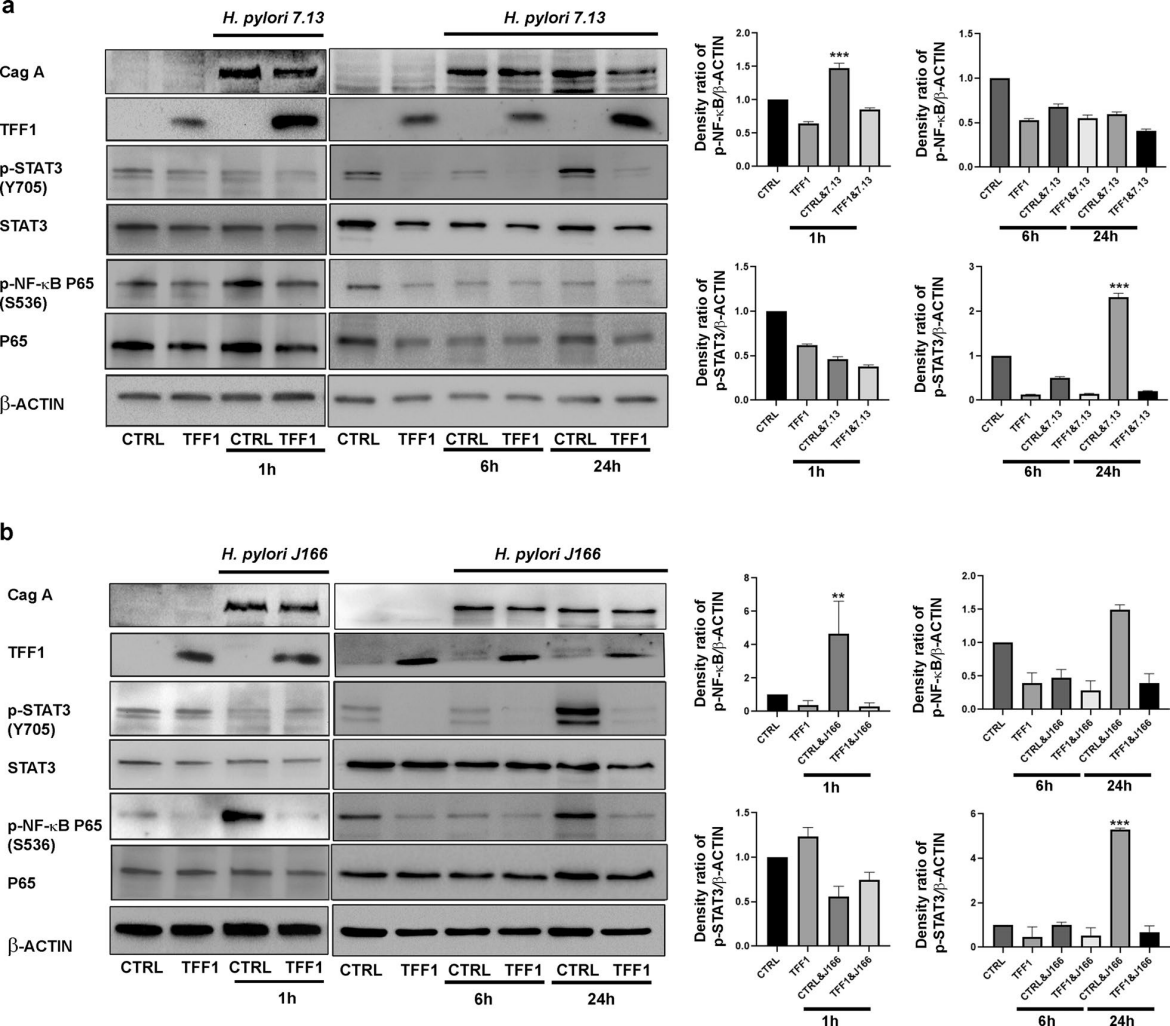
TFF1 attenuates *H. pylori-induced* activation of NF-κB and STAT3 in vitro. **a–b**) Western blot analysis of STAT3 and NF-κB in AGS cell lines stably expressing TFF1 or empty vector pcDNA infected with *H. pylori* J166 (**a**) or 7.13 (**b**) strains at different time points 1, 6 and 24 h. *H. pylori* increases of P-NF-κB-P65 (S536) and P-STAT3 (Y705) protein levels after 1 and 24 h of infection, respectively and reconstitution of TFF1 expression abolished this increase. β-ACTIN was used as a protein loading control, and TFF1 expression was confirmed using TFF1 specific antibody. The relative intensity ratio of p-STAT3 (y705)/β-Actin and p-NF-κB/β-Actin were calculated by the Image-Lab software from BioRad. The Western blot results are representative of three independent experiments. The results are expressed as mean ± SEM of at least three independent experiments using a two-tailed Student’s *t*-test

In this study, we investigated the role of *H. pylori* in regulating NF-κB and STAT3. We aimed to determine the order of activation. We also investigated the role of TFF1 in suppressing these signaling pathways. First, we used AGS human gastric cancer cell lines expressing TFF1 or empty-vector pcDNA as a control (CTRL). Cells were infected with *H. pylori* strain J166 or 7.13 and collected at 1, 6 and 24 h after infection. Our results demonstrated an early induction of p-NF-κB-P65 (Ser536) at 1 h, whereas p-STAT3 (Y705) was delayed and increased at 24 h time point, following infection with *H. pylori* strain 7.13 or J166. However, this increase was reduced with the reconstitution of TFF1 in AGS cells (Fig. 1a, b). To confirm this finding, we used two gastric cancer cell lines SNU-1 and HGC-27. SNU-1 cells were transiently transfected with TFF1-PTT5 or empty vector PTT5 for 48 h. HGC cells were treated with recombinant TFF1 (400 ng/ml) overnight. Next, the cells were infected with *H. pylori* strain 7.13 and then collected at 1, 6, and 24 h. SNU-1 cells demonstrated a similar pattern as found in AGS. We detected an increase of p-NF-κB-P65 (Ser536) at 1 h followed by an increase of p-STAT3 (Y705) at 24 h time point (Additional file 1: Figure S1a). Using recombinant TFF1 protein, HGC-27 cells showed an increase of p-NF-κB-P65 (Ser536) at 6 h, followed by increase of p-STAT3 (Y705) at 24 h time point (Additional file 1: Figure S1b). This increase of p-NF-κB-P65 (Ser536) and p-STAT3 (Y705) was abolished with the reconstitution of TFF1 protein in both SNU-1 and HGC-27 cells (Additional file 1: Figure S1a, b). These data suggest that the activation of NF-κB occurs first in response to *H. pylor*i infection followed by the activation of STAT3. TFF1 plays a negative role in regulating *H. pylori*-mediated activation of NF-κB and STAT3 in gastric cancer cells.

### TFF1 suppresses *H. pylori*–mediated STAT3 and NF-κB nuclear localization

**Fig. 2:**
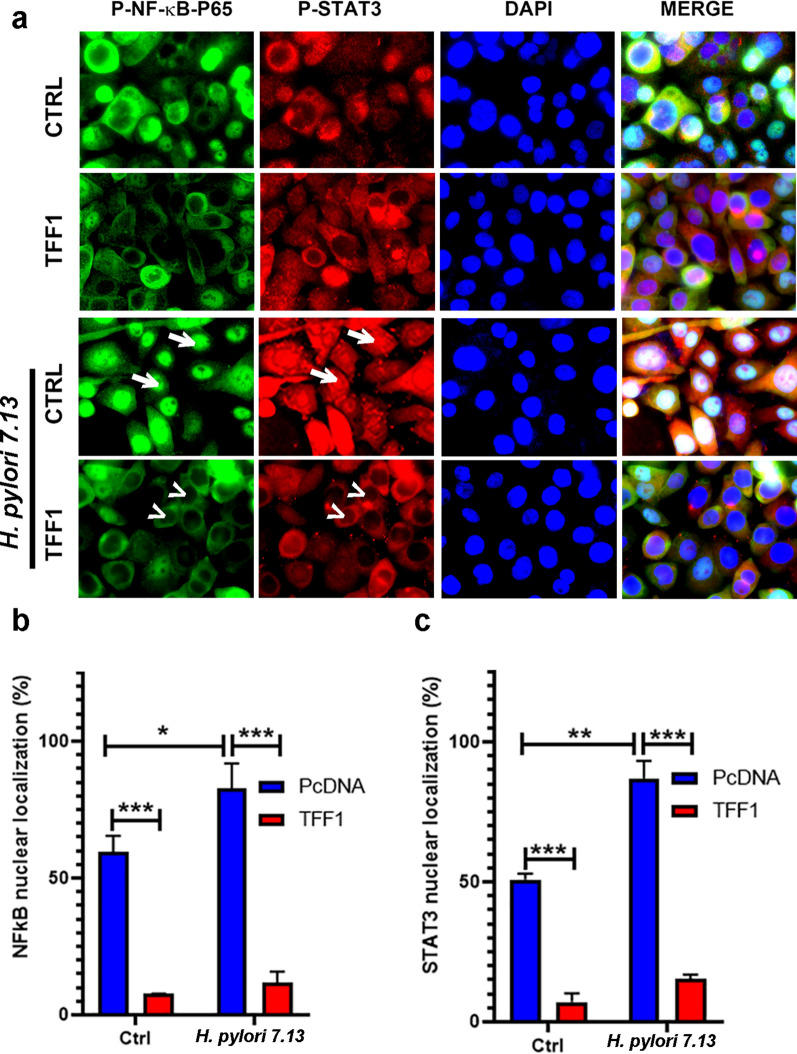
Reconstitution of TFF1 suppresses *H. pylori*-mediated nuclear localization of NF-κB and STAT3. (**a)** In vitro immunofluorescence assay showing nuclear localization of p-NF-κB–p65 and p-STAT3 in uninfected and *H. pylori* (7.13 strain) infected AGS stable cells. After infection, AGS-pcDNA cells demonstrated an increase of NF-κB–p65 (Green) and STAT3 (Red) nuclear localization (arrows). However, after reconstitution of TFF1, the percentage of nuclear staining of both NF-κB and STAT3 was decreased (arrowheads). DAPI (blue) was used as a nuclear counterstain. Original magnification, × 400. **b**–**c** The bar graphs are showing the quantification of nuclear NF-κB (**b**) and STAT3 (**c**) staining in at least 200 counted cells, presented as percentage ± SEM

To investigate the role of TFF1 in suppressing *H. pylori*-mediated activation of STAT3 and NF-κB, we performed an immunofluorescence assay. We used AGS cells stably expressing TFF1 or empty vector pcDNA for infection with *H. pylori* 7.13 and J166 for 24 h. Our results demonstrated that in AGS-pcDNA control cells, *H. pylori* infection induced a significant increase in the percentage of cells with nuclear NF-κB-P65 (Fig. 2a-b, P < 0.05; Additional file 2: Figure S2a, b, P < 0.01) and STAT3 (Fig. 2a-c; Additional file 2: Figure S2a–c, P < 0.01), as compared to uninfected cells. However, the expression of TFF1 significantly reduced *H. pylori*-induced nuclear translocation of NF-κB (Fig. 2a-b; Additional file 2: Figure S2a, b, P < 0.001) and STAT3 (Fig. 2a-c; Additional file 2: Figure S2a–c, P < 0.001) as compared to control cells. To confirm the immunofluorescence data, we examined the levels of p-NF-κB and p-STAT3 in cytosol and nuclear fractions by using western blot analysis. We used AGS cells stably-expressing TFF1 or empty vector pcDNA infected with *H. pylori* 7.13 at 1 and 24 h. The results showed an increase of p-NF-κB after 1 h infection and 24 h for p-STAT3 in the nuclear fraction after *H. pylori* infection. This increase was completely abolished after TFF1 reconstitution (Additional file 3: Figure S3). These data indicated that TFF1 suppresses *H.pylori*- induced nuclear translocation of NF-κB-P65 and STAT3.

### TFF1 abrogates *H. pylori*-induced STAT3 and NF-κB transcriptional activation

**Fig. 3:**
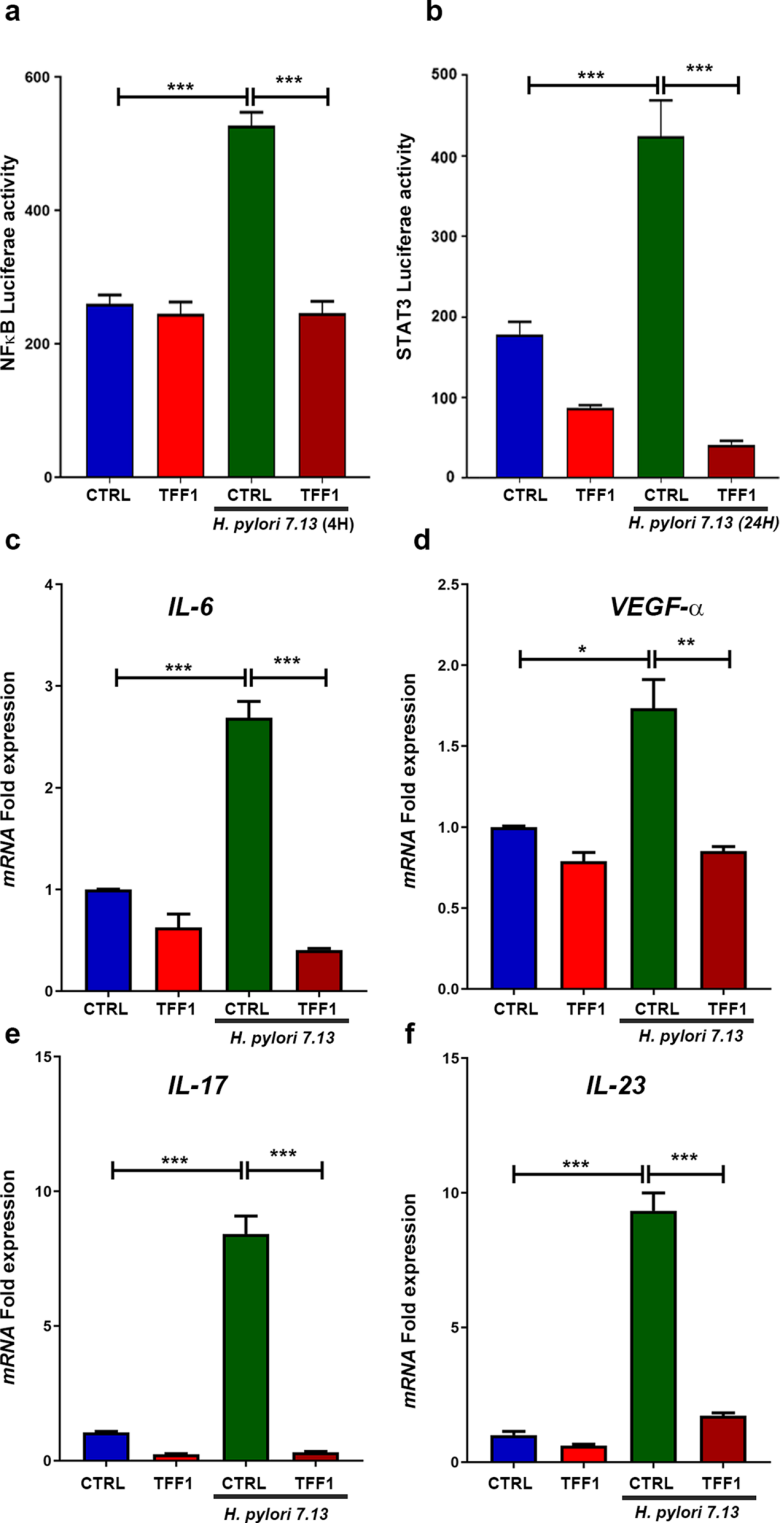
TFF1 expression alters *H. pylori*-induced transcriptional activation and regulation of NF-κB and STAT3 target genes. **a-b** Luciferase activity assay using NF-κB-Luc (**a**) and STAT3-Luc (**b**). AGS-pcDNA and AGS-TFF1 cells were co-transfected with NF-κB-Luc or STAT3-Luc and β-galactosidase plasmids. After 24 h, cells were infected with *H. pylori* 7.13 strain. Cells were collected 4 h after infection for NF-κB-Luc and 24 h for STAT3-Luc measurements.  *H. pylori* 7.13 infection of AGS-pcDNA cells significantly increased the luciferase activity, which was reduced after the reconstitution of TFF1. The bar graphs represent the mean ± SEM of 3 independent experiments. **c**–**f** RT-qPCR analysis showing a decrease in mRNA expression of pro-inflammatory target genes (*IL-6,VEGF-α, IL-17 and IL-23*) in AGS-TFF1 cells relative to AGS-pcDNA cells, following infection with *H. pylori* 7.13. The bars represent the mean ± SEM of three independent experiments

To confirm the effect of TFF1 on *H. pylori*-mediated activation of STAT3 and NF-κB, we checked the transcriptional activation of both transcriptional factors using a luciferase reporter assay. AGS stably-expressing TFF1 or empty vector pcDNA were transfected with pNF-κB-Luc or pSTAT3-Luc. The next day, cells were infected with *H. pylori* strains (7.13 or J166) for the duration of 4 h to measure the activity of NF-κB. The AGS-pcDNA control cells showed a significant increase in activation of pNF-κB-Luc (Fig. 3a-b; Additional file 4: Figure S4a, P < 0.001). However, the activation of STAT3 reporter was only obvious at 24 h post-infection with *H. pylori* (Fig. 3b; Additional file 4: Figure S4b, p < 0.001) confirming our earlier data in Fig. 1. In contrast, TFF1-expressing AGS cells  infected with *H. pylori* showed a significant decrease of NF-κB (Fig. 3a; Additional file 4: Figure 4a, P < 0.001) and STAT3 (Fig. 3b; Additional file 4: Figure 4b, P< 0.001) luciferase activities, compared to infected AGS-pcDNA control cells. Together, the reporter assays confirmed the immunofluorescence and Western blot results indicating that (1) NF-κB activation occurs at an earlier time point than STAT3, (2) TFF1 expression decreases *H. pylori*-mediated NF-κB and STAT3 activation in gastric cancer cells.

### TFF1 suppresses *H. pylori*-induced STAT3 and NF-κB target genes

NF-κB and STAT3 transcriptional factors can control an overlapping set of cytokines and pro-inflammatory genes [11, 37]. We assessed the mRNA expression level of *IL-6, VEGF-α, IL-17 and IL23*, following *H. pylori* infection, in conditions that included the reconstitution of TFF1. AGS cells stably-expressing TFF1 or empty vector pcDNA were infected with *H. pylori* 7.13 for 6 h. RT-qPCR results indicated a significant increase of mRNA expression of *IL-6* (P < 0.001), *VEGF-α* (P < 0.05), *IL-17* (P < 0.001), and *IL-23* (P < 0.001) in control cells after *H. pylori* infection, as compared with uninfected control cells (Fig. 3c–f). The reconstitution of TFF1 significantly reduced the mRNA expression of the prior genes, compared with their corresponding control cells after infection with *H. pylori* (Fig. 3c–f).

**Fig. 4:**
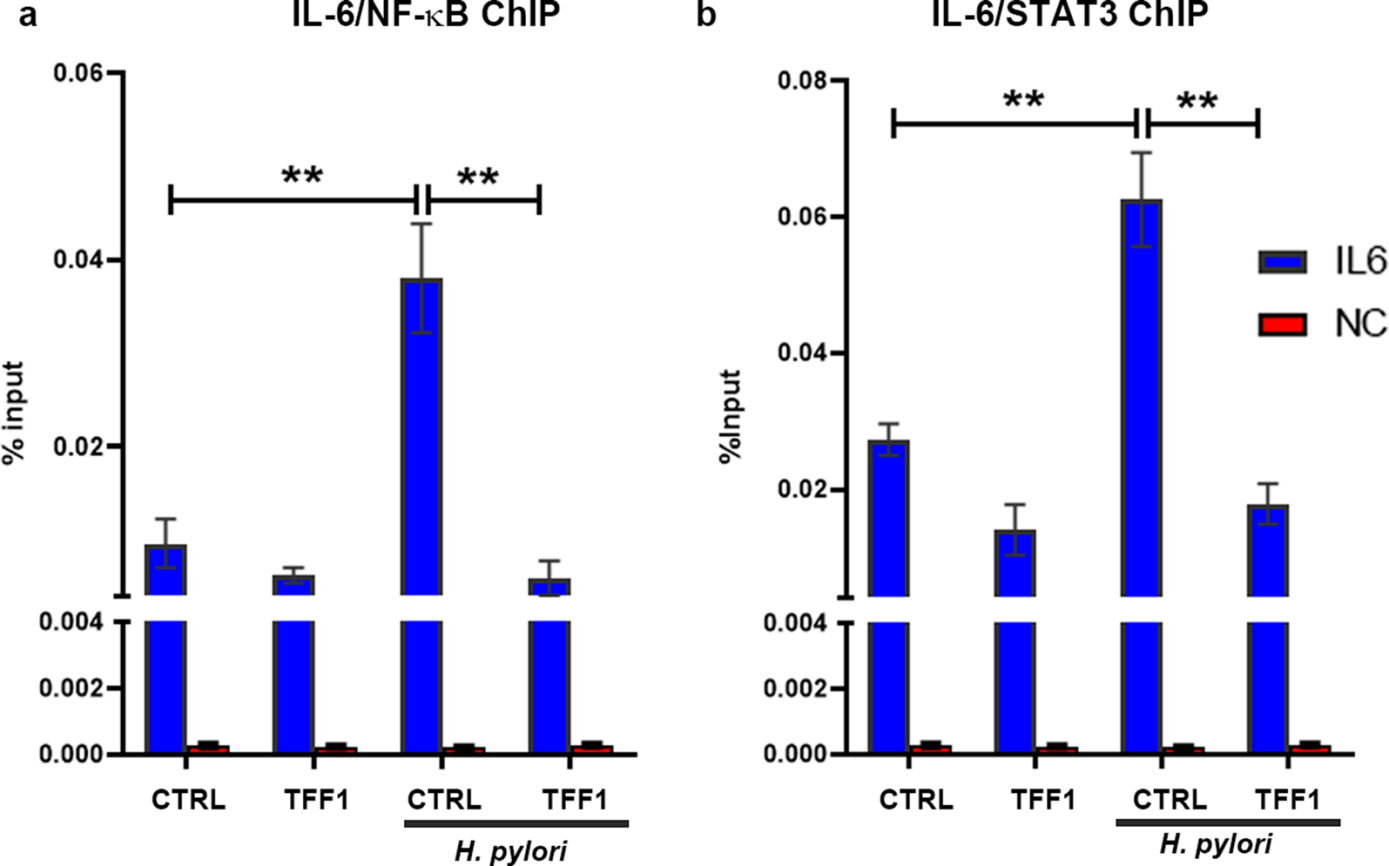
TFF1 abolishes *H. pylori*-induced increase of NF-κB and STAT3 binding to its target gene IL6. **a**–**b** ChIP assay in AGS-pcDNA and AGS-TFF1 stable cells infected with *H. pylori* (7.13) for a period of 4 h for NF-κB binding (**a**) and 24 h for STAT3 binding (**b**), followed by quantitative real-time PCR with primers designed for STAT3 and NF-κB binding site on *IL6* promoter regions. Control primers were designed 500 base pair upstream of STAT3 binding site on the *IL6* promoter. These primers were used as negative control (NC). Results are presented as a percentage of input

We next confirmed the role of TFF1 in suppressing the transcription activity by reducing the binding of NF-κB and STAT3 to their target genes. By using quantitative chromatin immunoprecipitation (ChIP) assay for IL6, a common target gene for NF-κB and STAT3, we detected a significant increase of NF-κB and STAT3 recruitment to IL-6 promoter (P < 0.01, Fig. 4a-b respectively) in AGS pcDNA cells control after *H. pylori* infection for 4 h for NF-κB-ChIP and 24 h for STAT3-ChIP. However this increase was significantly abolished following the reconstitution of TFF1 in AGS cells. Collectively, these results suggest that TFF1 suppresses  *H. pylori*-mediated NF-κB and STAT3 transcription activation and binding to and upregulation of pro-inflammatory target genes in gastric epithelial cells. These results are consistent with our Western blot findings (Fig. 1), indicating that activation of STAT3 occurs at a time point after NF-κB activation in conditions of *H. pylori*. Thus, the data suggest that activation of STAT3 in response to *H. pylori* infection may be dependent on the initial activation of NF-κB.

### *H. pylori*-mediated activation of STAT3 through NF-κB

**Fig. 5:**
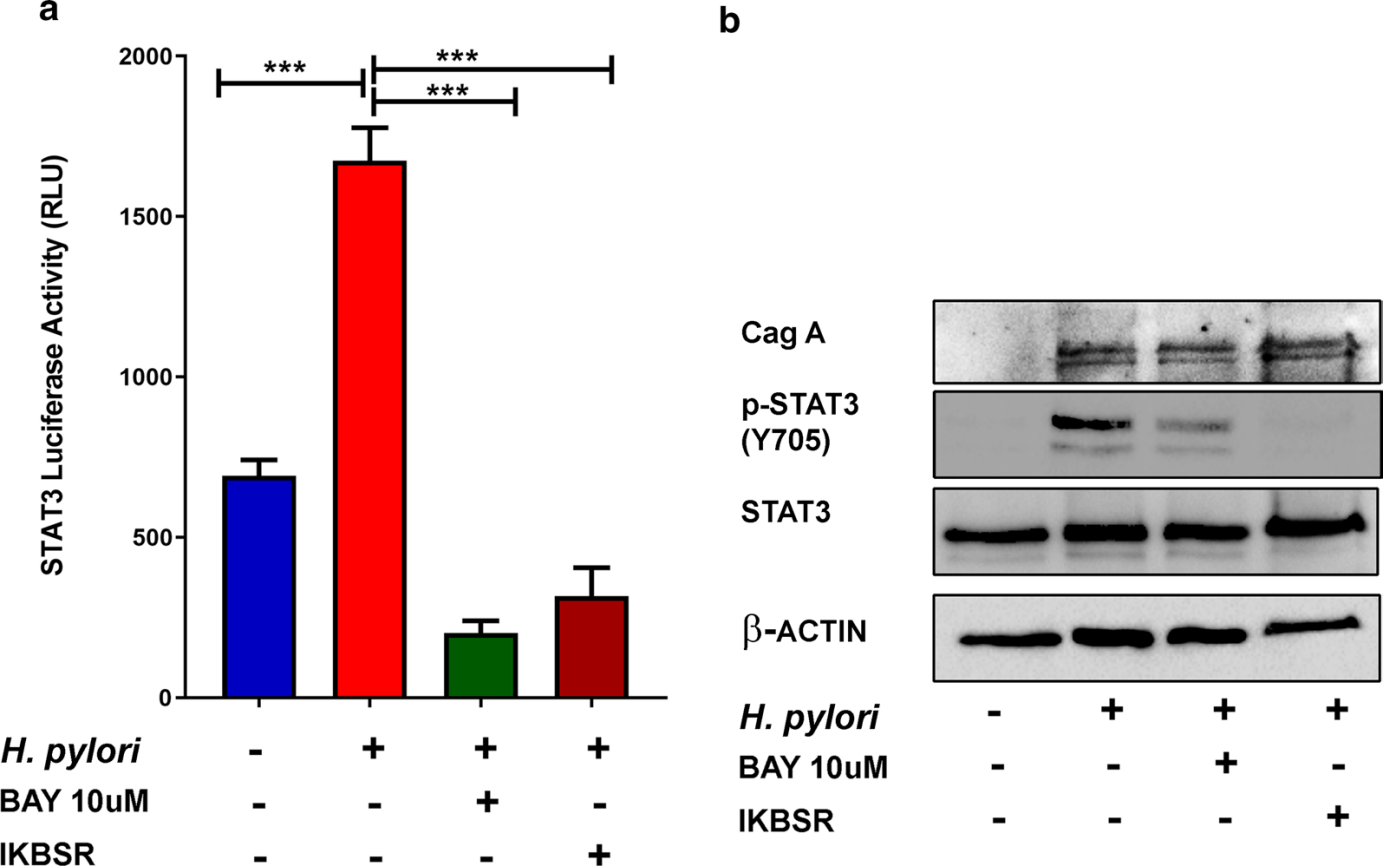
BAY and Iκ-B super repressor inhibit STAT3 activation. **a** The luciferase reporter assay using a STAT3-Luc (**a**) reporter plasmids. *H. pylori* infection of AGS-pcDNA cells significantly increased the luciferase activity, which was reduced after treatment with BAY (10 µM) or transfection with Iκ-B super-repressor plasmid The bar graphs represent the mean ± SEM of 3 independent experiments. **b** Western blot analysis of p-STAT3 in AGS-pcDNA cell lines infected with *H. pylori,* 7.13 treated with BAY, or transfected with Iκ-B super repressor (Iκ-BSR). The increase of p-STAT (Y705) protein level in *H. pylori*-infected cells was abolished after treatment with BAY or transfected with Iκ-BSR plasmid. The results are representative of three independent experiments

Our previous studies, showed that TFF1 suppresses *H. pylori* and TNF-α-mediated NF-κB activity through regulation of IKK pathway [23, 26]. Based on our results showing activation of NF-κB before STAT3 activation by *H. pylori* (Fig. 1), we hypothesized that, in some conditions such as *H. pylori* infection, STAT3 activation is dependent on NF-κB, where TFF1 suppresses STAT3 through inhibition of NF-κB pathway. To test this hypothesis, we used BAY 11–7082 (BAY), a specific NF-κB pathway inhibitor, or IκB super-repressor plasmid. For BAY, cells were treated overnight with BAY; the next day,  cells were infected with *H. pylori* and collected after 4 h for NF-κB and 24 h for STAT3 analysis. For IκB super-repressor, cells were first transfected with the plasmid,  and on the next day cells were infected with *H. pylori* and collected after 4 h for NF-κB and 24 h for STAT3 analysis. Infected and uninfected control cells were also collected at 4 and 24 h timepoints. Our data showed, as expected, a significant increase of NF-κB-Luc (Additional file 5: Figure S5a, P < 0.001) and STAT3-Luc (Additional file 5: Figure S5a, P < 0.01) activation after *H. pylori* infection with 7.13 strain. Both BAY and the Iκ-B super-repressor significantly inhibited the luciferase activity of NF-κB after *H. pylori* 7.13 infection (Additional file 5: Figure S5a, P  < 0.05 and P < 0.01). Of note, the STAT3-Luc activity was significantly reduced after treatment with BAY (10 µM) and completely abolished after transfection with Iκ-B super-repressor expression plasmid (Additional file 5: Figure S5a, P < 0.001). Using western blot, we confirmed our results and showed that BAY and the Iκ-B super-repressor completely abolished the expression of phospho- NF-κB (Additional file 5: Figure S5b) and phospho-STAT3 after *H. pylori* infection as compared to control cell infected by *H. pylori* only (Fig. 5b). These data indicated that STAT3 activation can be dependent on the activation of NF-κB in the context of *H. pylori* infection.

### NF-κB Inhibitors reverse the inflammatory phenotype in mouse gastric tissues

**Fig. 6:**
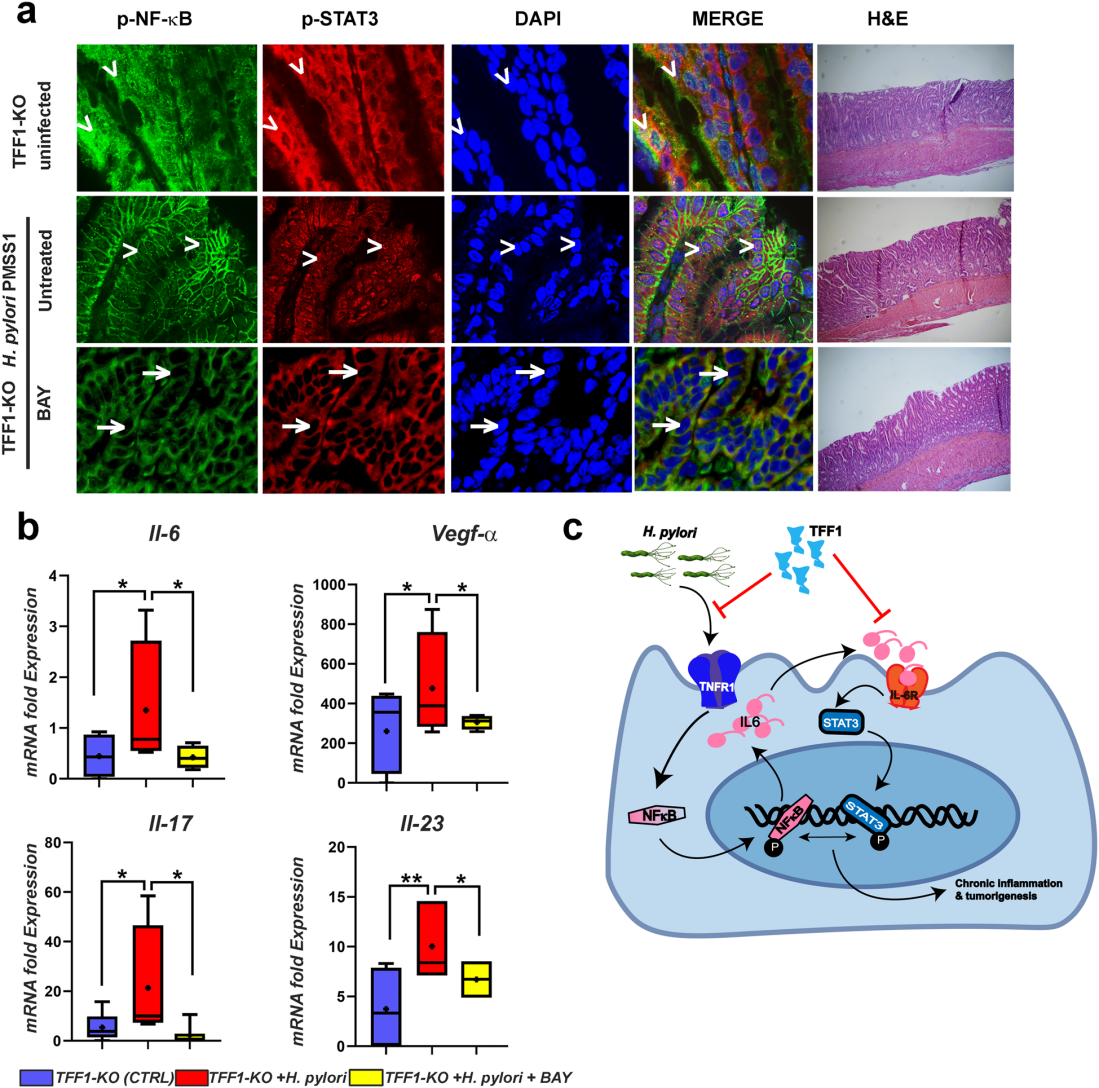
Treatment with STAT3 or NF-κB inhibitors reduced inflammation in TFF1-KO mice gastric tissues. **a** Immunofluorescence staining of phospho-NF-κB-P65 (Green) and phospho-STAT3 (Y705) (Red) in the antropyloric region of the glandular stomach of the TFF1-KO mice infected with PMSS1 *H. pylori* and treated or not by intraperitoneal injection with BAY (5 mg/Kg). In control, un-infected TFF1-KO showed more p-NF-κB-P65 and p-STAT3 nuclear staining after *H. pylori* infection (arrowheads). However, after treatment with BAY, staining showed reduced nuclear STAT3 and NF-κB (arrows). 4’, 6’ Diamino-2-phenylindole (DAPI) (blue) was used as a nuclear counterstain, original magnification (× 1000). **b** RT-qPCR analysis showing mRNA expression of pro-inflammatory target genes (*Il-6, Vegf-α, Il-17 and IL-23*) in *H. pylori*-infected TFF1-KO mice (10–12 weeks of age) treated or not with BAY (5 mg/Kg) and compared to TFF1-KO uninfected mice. Results are graphed using box-and-whisker blots to depict the smallest value, lower quartile, median, upper quartile, and largest value. (●) Indicate the mean. **c** A schematic cartoon depicting the role of TFF1 in regulating the inflammation in gastric epithelial cell through inhibition of NF-κB-mediated activation of STAT3 in response to *H. pylori*

Our previous reports demonstrated that loss of TFF1 induces activation of STAT3 and NF-κB target genes [23, 33]. Therefore, we investigated whether the inhibition of NF-κB could affect STAT3 and reverse *H. pylori*-induced inflammation after TFF1 loss in mouse gastric tissues. TFF1 knockout mice were infected with 3 doses of *H. pylori* (PMSS1 strain). After 2 weeks, mice received three doses of BAY 11–7082 (NF-κB inhibitor) (5 mg/kg), one dose every three days. After the last dose,  the *H. pylori* infected and non-infected mice were sacrificed. To confirm the inhibition of STAT3 and NF-κB activity by BAY, we performed immunofluorescence using p-STAT3 (Y705) and p-NF-κB-P65 (Ser536) specific antibodies. Our data indicated an increase in nuclear co-localization of STAT3 and NF-κB after *H. pylori* infection in mouse gastric tissues (Fig. 6a), compared with uninfected mice. After treatment with BAY, the nuclear co-localization of STAT3 and NF-κB was completely abolished in TFF1-KO mouse gastric tissue (Fig. 6a). Next, we examined the mRNA expression of the pro-inflammatory genes (*Il-6, Vegf-α, Il17 and Il-23*), a well-known readout for both STAT3 and NF-κB pathways [38]. The RT-qPCR data demonstrated a significant increase in mRNA expression of *Il-6 (P* < *0.05), Vegf-α (P* < *0.05), Il17 (P* < *0.05), and Il-23* (*P* < *0.01*) in mice infected with *H. pylori*, as compared with uninfected control mice (Fig. 6b). At the same time, the treatment with BAY significantly reduced the mRNA expression of the genes mentioned earlier after *H. pylori*-infection, compared with non-treated *H. pylori* infected mice (Fig. 6b P < 0.05). A cartoon summarizing our findings is shown in Fig. 6c.

## Discussion

The strong association between chronic inflammation and cancer development is attributed to a number of causal factors, including infectious agents such as bacteria, viruses, and parasites [39, 40]. Chronic inflammatory conditions create an inflammatory microenvironment that is more susceptible to tumorigenesis [41, 42]. NF-κB and STAT3 are two major inflammatory pathways that promote the initiation and progression of several cancers, including gastric cancer [43]. In this study, we investigated the role of *H. pylori*, a spiral and gram-negative bacterium, in activation of NF-κB and STAT3 signaling pathways. We demonstrate that activation of NF-κB is an early event that precedes activation of STAT3. We found that activation of STAT3 by *H. pylori* is dependent on activation of NF-κB, where inhibition of NF-κB by pharmacologic or genetic means abrogates both NF-κB and STAT3 activity.

Infection with *H. pylori* is the main risk factor for chronic inflammation and gastric cancer [44, 45]. Earlier reports have shown that *H. pylori* promotes activation of NF-κB and STAT3 pro-inflammatory signaling [46, 47]. *H. pylori* strains carrying the cytotoxin-associated antigen (CagA) activate NF-κB and STAT3, through phosphorylation [48–51]. Once STAT3 and NF-κB are phosphorylated, they translocate to the nucleus and promote the expression of  several inflammatory target genes [52]. Our study aimed to investigate both NF-κB and STAT3 to determine if there is a possible causal relationship between activation of these two important pathways, in response to infection with *H. pylori.* Our data confirmed nuclear localization of STAT3 and NF-κB, indicating their activation after infection with J166 and 7.13 *H. pylori* strains. We also detected up-regulation of several target genes such as *IL-6*, *VEGF-α*, *IL-17* and *IL-23*, both in vitro and in vivo. Surprisingly, in contrast to the rapid activation of NF-κB at one hour, STAT3 activation was delayed post-infection and reached its peak at 24 h. Using BAY 11–7082 or IκB super-repressor to inhibit NF-κB, we detected abrogation of NF-κB activation, and STAT3, in response to *H. pylori* infection. Previous reports suggested that STAT3 can be activated by several NF-κB target genes, including IL-6 [53]. Therefore, we hypothesized that the rapid NF-κB activation by *H. pylori* induces IL6 expression, which serves in an autocrine loop to mediate a delayed activation of STAT3. Using ChIP assays, we confirmed that NF-κB and STAT3 bind to the IL6 promoter, suggesting that activation of NF-κB initiates a sustained circuit of feedback and autocrine activation of both NF-κB and STAT3.

The expression of TFF1 is frequently downregulated during gastric tumorigenesis through several molecular mechanisms, including deletion, promoter methylation, and transcription regulation [18–22]. We have previously shown that TFF1 suppresses IL6-mediated activation of STAT3 by interfering with the IL6 receptor complex [33]. Our present findings add to this complexity by showing that TFF1 interferes with IL6R complex [33],  and suppresses IL6 induction by NF-κB, thereby inhibits NF-κB mediated activation of STAT3 in response to *H. pylori*. We have previously shown that TFF1 can suppress NF-κB by interfering with the TNFα receptor complex [23]. The activation of NF-κB and STAT3 can collaboratively mediate the expression of several overlapping inflammatory target genes [11, 42]. Our data demonstrated upregulation of several pro-inflammatory target genes in vivo and in vitro, following *H. pylori* infection. The reconstitution of TFF1 reversed these effects and abrogated induction of *IL-6, VEGF-α, IL-17* and *IL-23* by *H. pylori* infection. Thus, our results demonstrate that TFF1 is capable of suppressing *H. pylori*-mediated activation of both signaling pathways, where NF-κB appears to be an important initial driving factor for activation of this autocrine pro-inflammatory signaling loop.

## Conclusion

Our findings indicate that *H. pylori* infection mediates an early activation of NF-κB with induction of IL6 that subsequently promotes a delayed autocrine activation of STAT3. The frequently reported loss of TFF1 during gastric tumorigenesis could be a critical step in promoting *H. pylori*-mediated activation of NF-κB—STAT3 signaling circuit. This discovery suggests that novel therapeutics that target the initiation of inflammation may effectively reduce the progression of *H. pylori* mediated gastric carcinogenesis cascade.

## Supplementary Information

**Additional file 1: Figure S1**. TFF1 abolished H.pylori-induced activation of NF-κB and STST3 in gastric cancer cell lines.

**Additional file 2: Figure S2**. Reconstitution of TFF1 suppresses H.pylori-mediated nuclear localization of NF-κB and STAT3.

**Additional file 3: Figure S3**. TFF1 decreases H.pylori induced nuclear expression of p-NF-κB and p-STAT3 in vitro.

**Additional file 4: Figure S4**. TFF1 expression alters H.pylori-induced transcriptional activation of NF-κB and STAT3.

**Additional file 5: Figure S5**. The luciferase reporter assay using a NF-κB-Luc (a) reporter plasmids.

## Data Availability

The data that support the findings of this study are available from the corresponding author upon reasonable request.

## Abbreviations

TFF1: Trefoil factor 1
*H. pylori*: *Helicobacter pylori*
NF-κB: Nuclear factor kappa-light-chain-enhancer of activated B cells
STAT3: Signal transducer and activators of transcription
PBS: Phosphate buffered saline
