# Supplementary figures and images for: NF-kB-dependent activation of STAT3 by H. pylori is suppressed by TFF1
Source: Cancer Cell Int. 2021 Aug 21;21:444. doi: 10.1186/s12935-021-02140-2 (PMC8380333; doi:10.1186/s12935-021-02140-2)

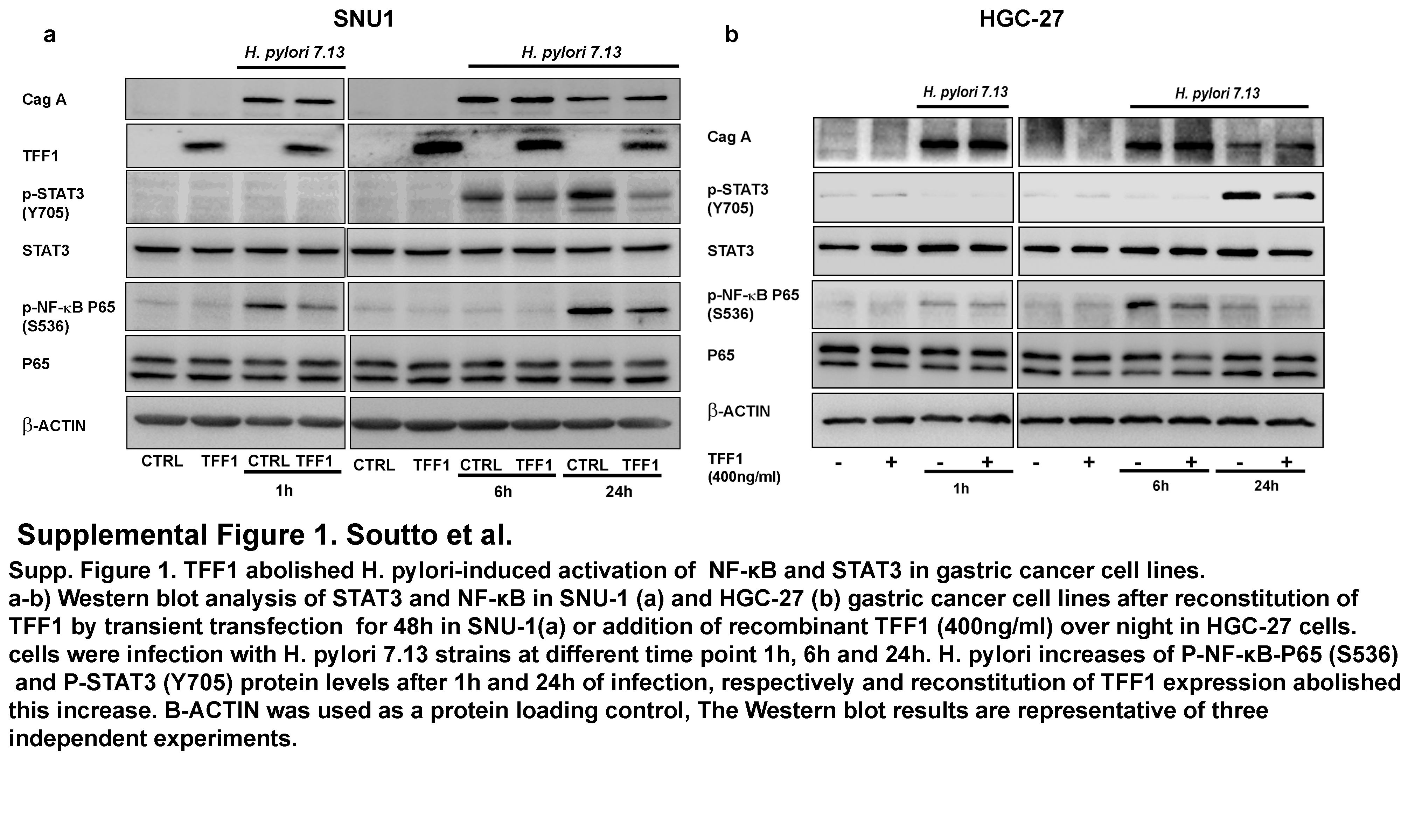

Supplement: Supplementary file 1 — Additional file 1: Figure S1. TFF1 abolished H.pylori-induced activation of NF-κB and STST3 in gastric cancer cell lines. [file 12935_2021_2140_MOESM1_ESM.tif]

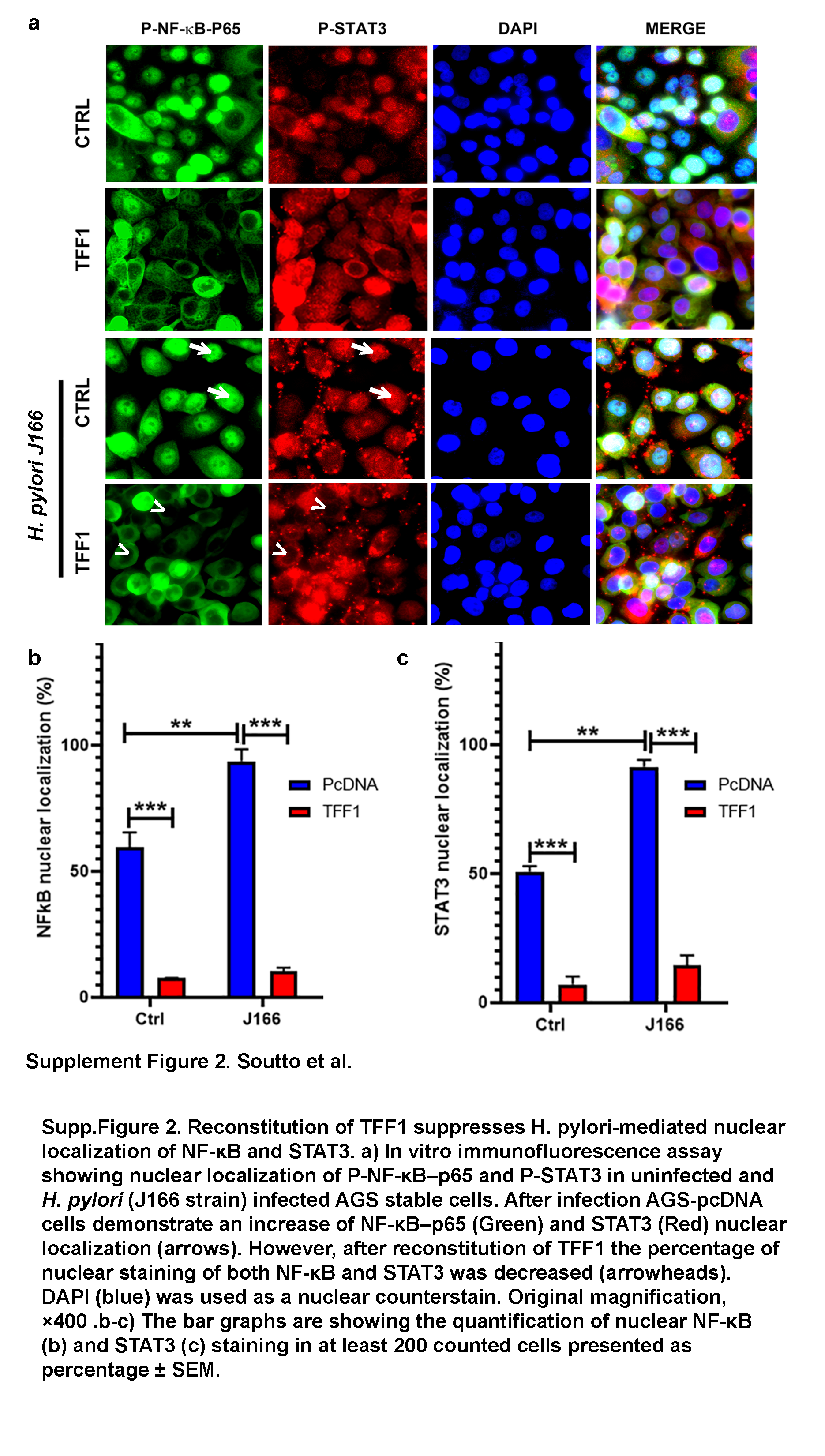

Supplement: Supplementary file 2 — Additional file 2: Figure S2. Reconstitution of TFF1 suppresses H.pylori-mediated nuclear localization of NF-κB and STAT3. [file 12935_2021_2140_MOESM2_ESM.tif]

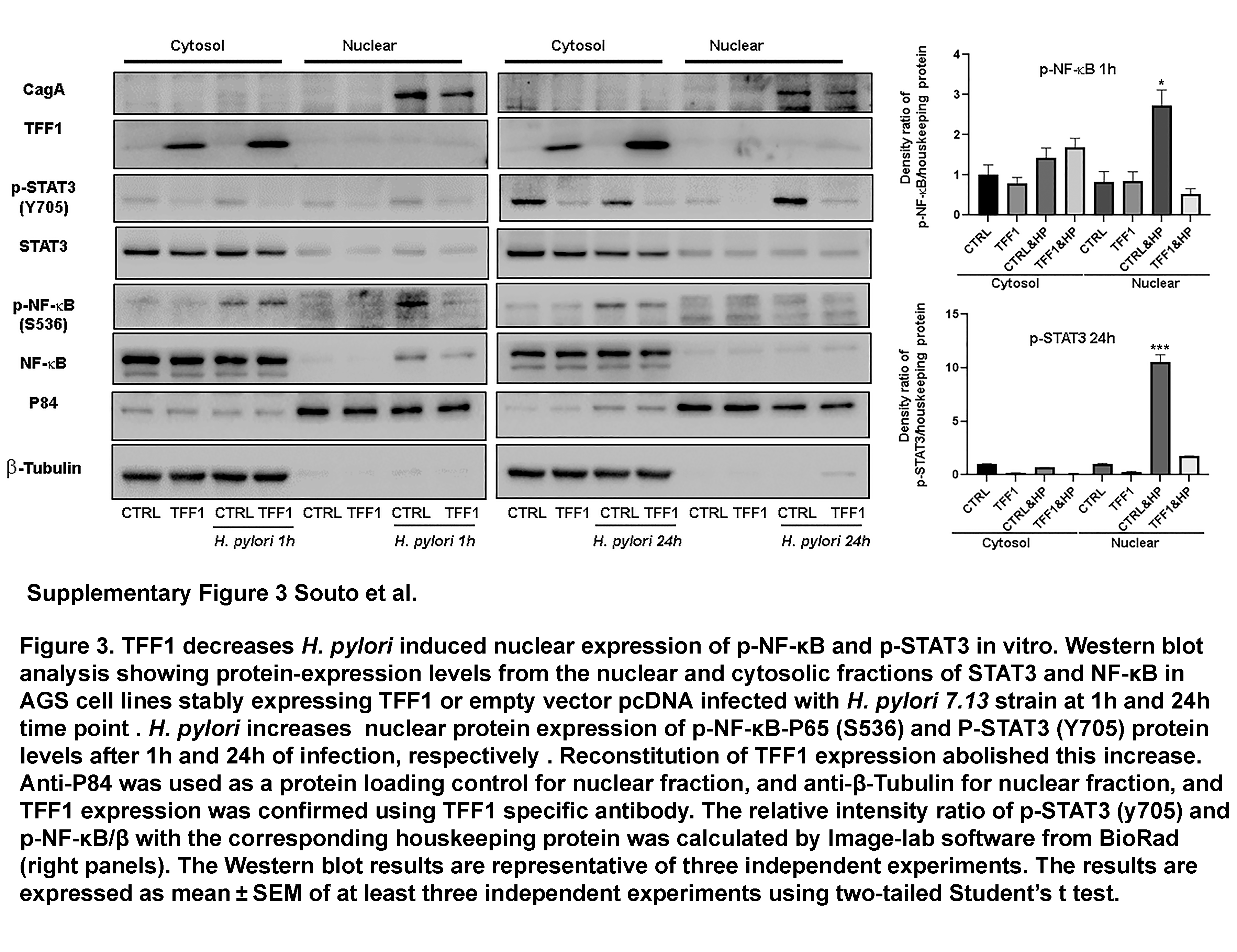

Supplement: Supplementary file 3 — Additional file 3: Figure S3. TFF1 decreases H.pylori induced nuclear expression of p-NF-κB and p-STAT3 in vitro. [file 12935_2021_2140_MOESM3_ESM.tif]

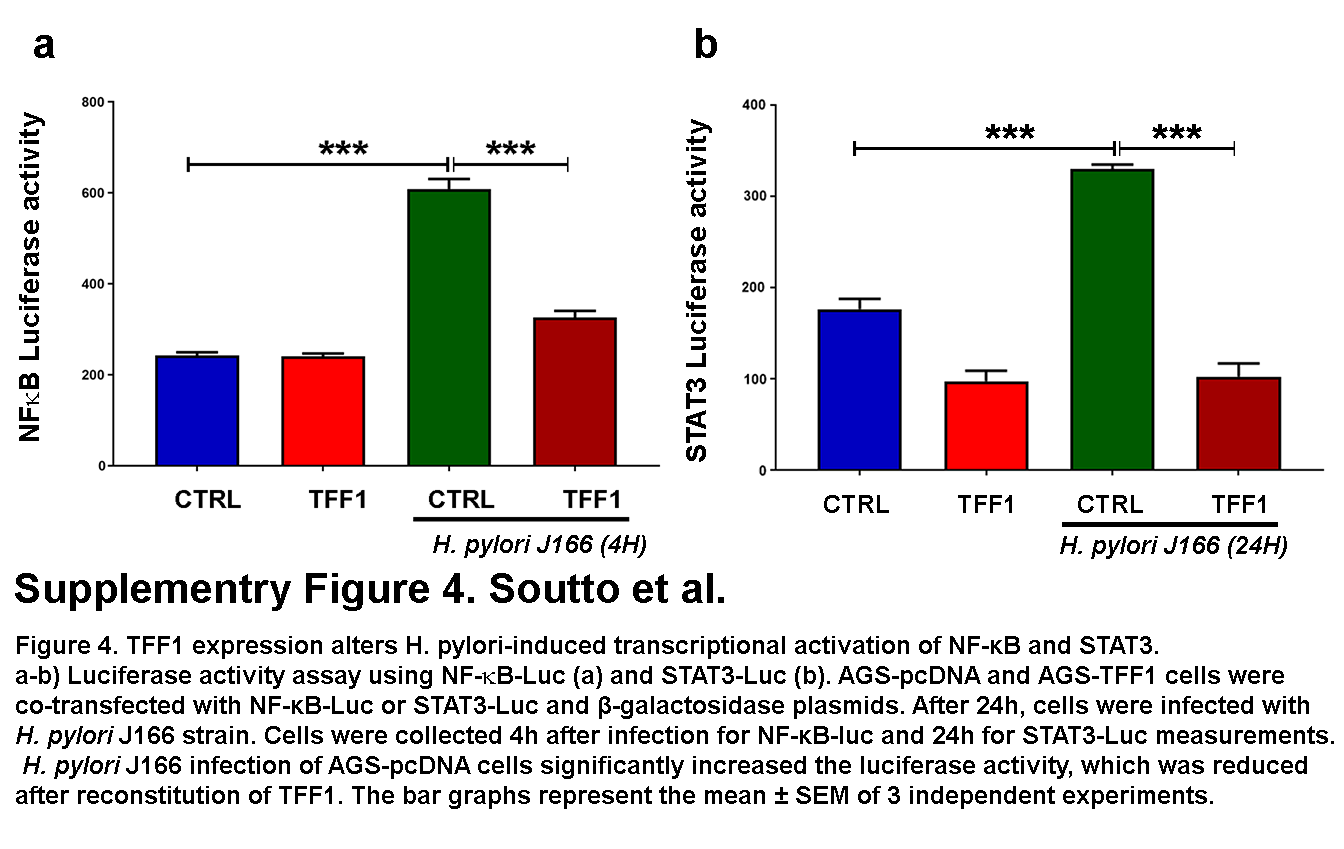

Supplement: Supplementary file 4 — Additional file 4: Figure S4. TFF1 expression alters H.pylori-induced transcriptional activation of NF-κB and STAT3. [file 12935_2021_2140_MOESM4_ESM.tif]

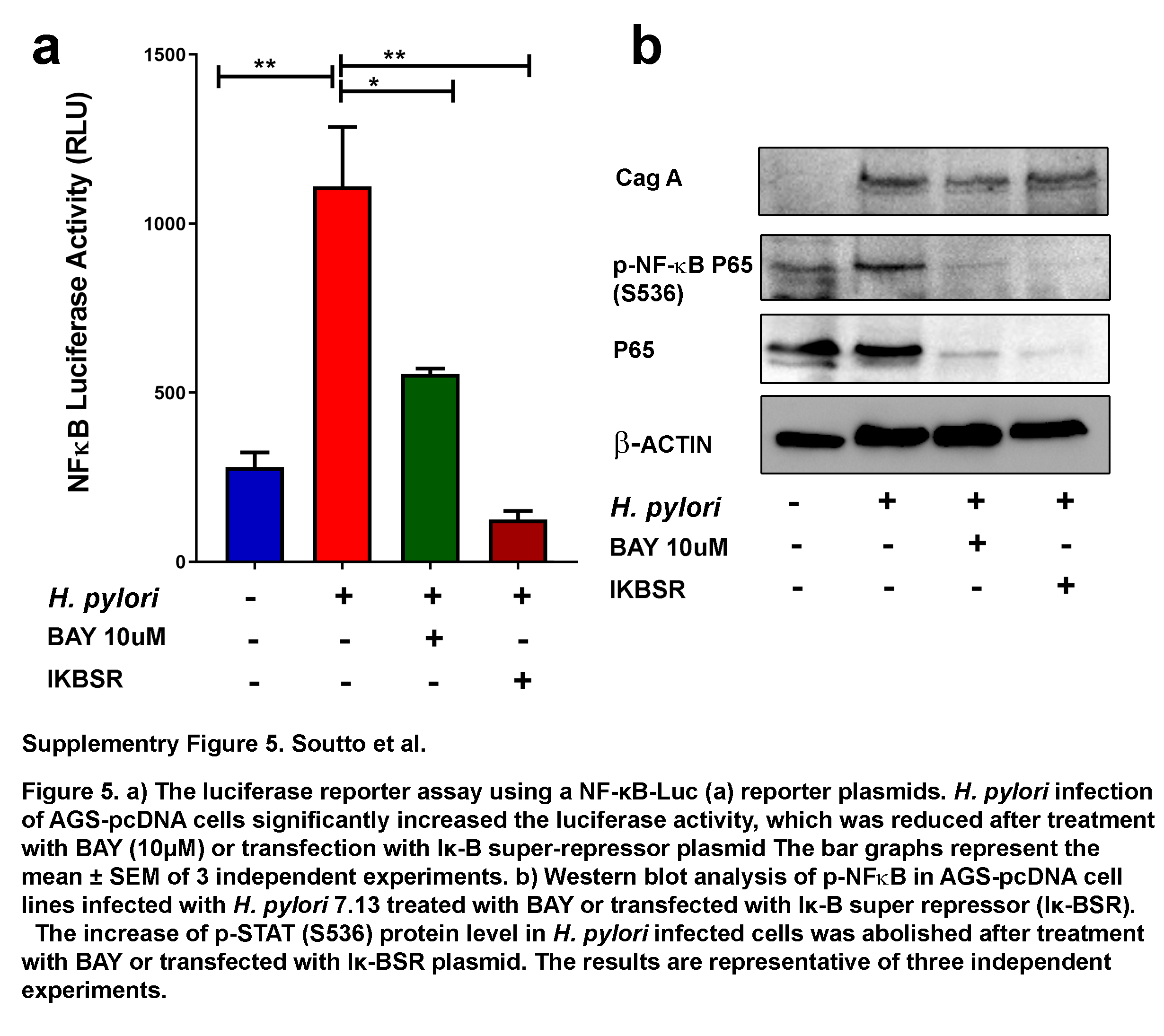

Supplement: Supplementary file 5 — Additional file 5: Figure S5. The luciferase reporter assay using a NF-κB-Luc (a) reporter plasmids. [file 12935_2021_2140_MOESM5_ESM.tif]
